# (2*RS*,5′*RS*)-3′,4′-Bis(4-chloro­phen­yl)-3,4-dihydro­spiro­[acridine-2,5′(4′*H*)-[1,2]oxazol]-1(2*H*)-one

**DOI:** 10.1107/S1600536812045084

**Published:** 2012-11-07

**Authors:** Ponmudisettu Narayanan, Shanmugavel Uma Maheswari, Krishnan Sethusankar

**Affiliations:** a Department of Physics, RKM Vivekananda College (Autonomous), Chennai 600 004, India; b Department of Chemistry, School of Organic Chemistry, Madurai Kamaraj University, Madurai 625 021, India

## Abstract

The title compound, C_27_H_18_Cl_2_N_2_O_2_, represents a racemic mixture of the corresponding *R*,*R* and *S*,*S* diastereomers. The isoxazoline ring adopts an envelope conformation with the spiro C atom deviating by 0.093 (2) Å from the rest of the ring. The six-membered keto-substituted carbocycle has a sofa conformation with the methyl­ene C atom adjacent to the spiro center deviating by 0.289 (2) Å from the mean plane of the remaining atoms. In the crystal, mol­ecules are linked *via* C—H⋯Cl inter­actions and C—Cl⋯O halogen bonds [2.958 (2) Å, 171.39 (7)°], which generate bifurcated *R*_2_^1^(6) ring motifs resulting in *C*_2_^1^[*R*_2_^1^(6)] chains running parallel to [010].

## Related literature

For the uses and biological importance of acridines, see: Asthana *et al.* (1991 ▶); Di Giorgio *et al.* (2005 ▶); Talacki *et al.* (1974 ▶). For related structures, see: Sridharan *et al.* (2009 ▶); Trzybiński *et al.* (2010 ▶). For graph-set notation, see: Bernstein *et al.*(1995 ▶)
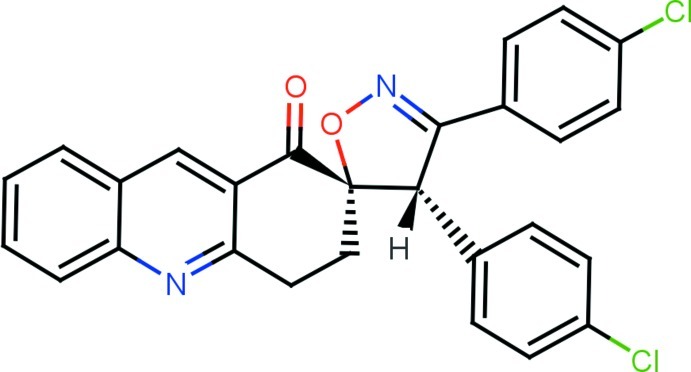


## Experimental

### 

#### Crystal data

C_27_H_18_Cl_2_N_2_O_2_
*M* *_r_* = 473.33
Monoclinic, 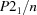

*a* = 7.6493 (4) Å
*b* = 15.1553 (7) Å
*c* = 19.4802 (8) Å
β = 90.392 (1)°
*V* = 2258.24 (18) Å^3^
*Z* = 4
Mo *K*α radiation
μ = 0.32 mm^−1^
*T* = 293 K
0.35 × 0.30 × 0.25 mm

#### Data collection

Bruker Kappa APEXII CCD diffractometer
Absorption correction: multi-scan (*SADABS*; Bruker, 2008 ▶) *T*_min_ = 0.895, *T*_max_ = 0.924
26147 measured reflections
5960 independent reflections
4189 reflections with *I* > 2σ(*I*)
*R*_int_ = 0.026

#### Refinement

*R*[*F* ^2^ > 2σ(*F* ^2^)] = 0.051
*wR*(*F* ^2^) = 0.143
*S* = 1.02
5960 reflections
298 parameters
H-atom parameters constrained
Δρ_max_ = 0.51 e Å^−3^
Δρ_min_ = −0.66 e Å^−3^

### 

Data collection: *APEX2* (Bruker, 2008 ▶); cell refinement: *SAINT* (Bruker, 2008 ▶); data reduction: *SAINT*; program(s) used to solve structure: *SHELXS97* (Sheldrick, 2008 ▶); program(s) used to refine structure: *SHELXL97* (Sheldrick, 2008 ▶); molecular graphics: *ORTEP-3* (Farrugia, 1997 ▶); software used to prepare material for publication: *SHELXL97* and *PLATON* (Spek, 2009 ▶).

## Supplementary Material

Crystal structure: contains datablock(s) global, I. DOI: 10.1107/S1600536812045084/ld2076sup1.cif

Structure factors: contains datablock(s) I. DOI: 10.1107/S1600536812045084/ld2076Isup2.hkl

Additional supplementary materials:  crystallographic information; 3D view; checkCIF report

## Figures and Tables

**Table 1:** Hydrogen-bond geometry (Å, °)

*D*—H⋯*A*	*D*—H	H⋯*A*	*D*⋯*A*	*D*—H⋯*A*
C10—H10*A*⋯Cl2^i^	0.97	2.78	3.681 (2)	154

Symmetry code: (i) 
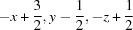
.

**Fig. 1.**
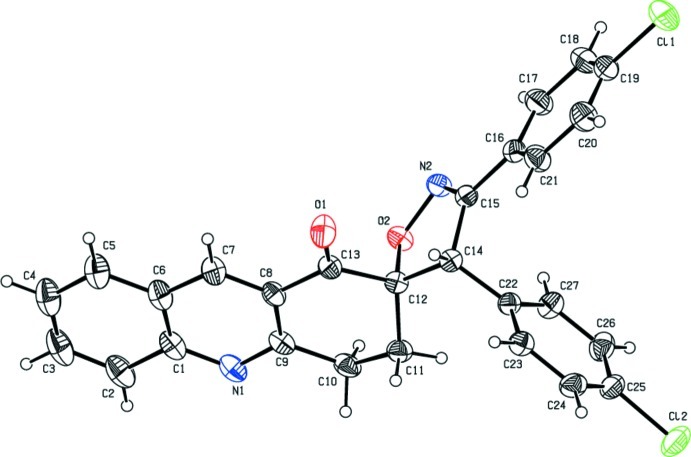
The molecular structure of the title compound with the atom numbering scheme. The displacement ellipsoids are drawn at 30% probability level.

**Fig. 2.**
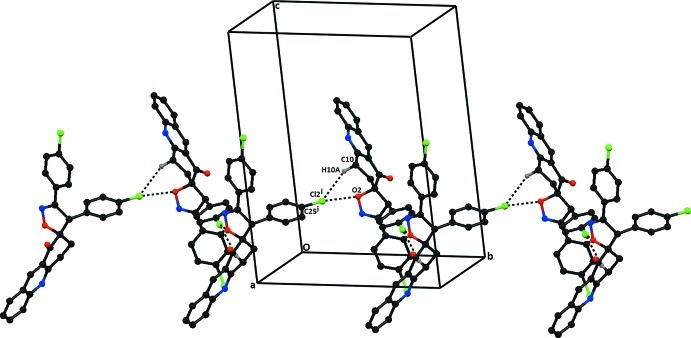
The crystal packing of the title compound, viewed along c-axis, showing bifurcated bonding including Cl2: C10—H10A···Cl2i hydrogen bonds and C25i—Cl2i···O2 halogen bonds, resulting into supramolecular chains C12[R12(6)] running parallel to [0 1 0] direction. The hydrogen atoms not involved in the hydrogen bonding have been excluded for clarity. The symmetry code: (i). 3/2 - x,-1/2 + y,1/2 - z.

## supplementary crystallographic information

### Comment

Acridine derivatives are biologically important compounds, which are found to possess mutagenic, antitumour (Talacki *et al.*, 1974), antibacterial, antiamoebic, hypersensitive, antiinflammatory and antiimplantation (Asthana *et al.*, 1991) activities. Also, they have been shown to exert toxicity towards plasmodium, trypanosoma and leishmania parasites (Di Giorgio *et al.*, 2005). Against this backround, X-ray study of the title compound has been carried out to study its structural aspects.

The title compound C_27_ H_18_ Cl_2_ N_2_ O_2_, consists of an isoxazole ring *spiro* attached to acridine ring system and the phenyl rings (C22–C27) and (C16–C21). X-ray analysis confirms the molecular stereochemistry as illustrated in Fig. 1. The quinoline ring system is essentially planar with a maximum deviation of -0.049 (2) Å for C9 atom. The quinoline ring system forms a dihedral angle of 86.12 (10) ° with the chlorophenyl ring (C16–C21), which shows that they are almost orthogonal to each other. The chlorophenyl ring (C22–C27) forms a dihedral angle of 74.99 (9) ° with the quinoline ring system.

The six membered carbocyclic ring (C8–C13) adopts a sofa conformation with the deviation of C11 by -0.289 (2) Å from the mean plane formed by the remaining ring atoms. The mean plane of the six membered ring (C8–C13) forms a dihedral angle of 85.34 (11) ° with the chlorophenyl ring (C16–C21), which shows that they are almost perpendicular to each other. The oxygen atom (O1) significantly deviates from the mean plane of the ring (C8–C13) by -0.3368 (18) Å. The mean plane of the six membered carbocyclic ring (C8–C13) forms a dihedral angle of 2.53 (8) ° with the quinoline ring system, which shows that they are almost coplanar.

The isoxazole ring (N2/O2/C12/C14/C15) adopts an envelope conformation with a maximum deviation of C12 by 0.0935 (18) Å from the mean plane formed by the remaining ring atoms. The isoxazole ring forms the dihedral angles of 82.77 (8) °, 83.52 (9) ° and 83.45 (10) ° with the quinoline bicyclic ring system, the six membered ring (C8–C13) and the phenyl ring (C22–C27), respectively. The chlorine atoms (Cl1 & Cl2) are significantly deviate from the phenyl ring (C16–C21) and (C22–C27) by -0.0113 (19) Å and 0.0666 (8) Å, respectively. The title compound exhibits structural similarities with already reported related structures (Sridharan *et al.*, 2009; Trzybiński *et al.*, 2010).

In the crystal, the molecules are linked *via* C10—H10A···Cl2^i^ intermolecular interaction and C25^i^—Cl2^i^···O2 halogen bonding (XB), between the chlorine atom(Cl2) and keto group oxygen atom(O2) of the acridine ring system [Cl2^i^···O2 = 2.958 (2) Å and a nearly linear C25^i^—Cl2^i^···O2 angle of 171.39 (7) °], which generate bifurcated *R*_2_^1^(6) ring motifs resulting in chains with the full graph-set designation of *C*_2_^1^[*R*_2_^1^(6)] running parallel to [0 1 0] axis. (Bernstein *et al.*, 1995) (Fig. 2). The symmetry code: (i). 3/2 - *x*,-1/2 + *y*,1/2 - *z*

### Experimental

A mixture of 3,4-dihydroacridin-1(2*H*)-one(200 mg, 1 mmol), 4-chloro benzaldehyde (168 mg, 1.2 mmol) and KOH (84 mg, 1.5 mmol) in DME (3 ml) was stirred at ambient temperature for 30 min. Then, 4-chloro-*N*-hydroxybenzimi doyl chloride (285 mg, 1.5 mmol) was added subsequently to the reaction mixture and stirred at room temperature for 10–12 h. The progress of the reaction was monitored by thin-layer chromatography with petroleum ether-ethylacetate (4:1 *v*/*v*) mixture as eluent. After completion of the reaction, the reaction mixture was extracted with ethyl acetate (2x20 ml), washed with water (2x10 ml), dried over anhydrous Na_2_SO_4_ and concentrated under reduced pressure. The residue thus obtained recrystallized from diethyl-ether to afford (2S,3'S)-3',4'-bis(4-chlorophenyl)-3,4-dihydro-1H,3'H-spiro[acridine-2,2'-[1,5]oxazole]-1-one as off white solid. Single crystals suitable for X-ray diffraction were prepared by slow evaporation of a solution of the title compound in ethanol at room temperature.

Yield = 89%,

m.p. = 214–216 °C.

### Refinement

The positions of the hydrogen atoms were localized from the difference electron density maps and the distances were geometrically constrained. The H atoms bound to the C atoms, with d(C—H) = 0.93 Å and *U*_iso_(H) = 1.2*U*_eq_(C) for aromatic, d(C—H) = 0.98 Å and *U*_iso_(H) = 1.2*U*_eq_(C) for methine, d(C—H) = 0.97 Å and *U*_iso_(H) = 1.2*U*_eq_(C) for methylene groups.

### Crystal data

**Table:**

C_27_H_18_Cl_2_N_2_O_2_	*F*(000) = 976
*M**_r_* = 473.33	*D*_x_ = 1.392 Mg m^−^^3^
Monoclinic, *P*2_1_/*n*	Mo *K*α radiation, λ = 0.71073 Å
Hall symbol: -P 2yn	Cell parameters from 5960 reflections
*a* = 7.6493 (4) Å	θ = 2.5–29.1°
*b* = 15.1553 (7) Å	µ = 0.32 mm^−^^1^
*c* = 19.4802 (8) Å	*T* = 293 K
β = 90.392 (1)°	Block, colourless
*V* = 2258.24 (18) Å^3^	0.35 × 0.30 × 0.25 mm
*Z* = 4	

### Data collection

**Table:**

Bruker Kappa APEXII CCD diffractometer	5960 independent reflections
Radiation source: fine-focus sealed tube	4189 reflections with *I* > 2σ(*I*)
Graphite monochromator	*R*_int_ = 0.026
ω and φ scans	θ_max_ = 29.1°, θ_min_ = 2.5°
Absorption correction: multi-scan (*SADABS*; Bruker, 2008)	*h* = −10→8
*T*_min_ = 0.895, *T*_max_ = 0.924	*k* = −19→20
26147 measured reflections	*l* = −26→26

### Refinement

**Table:**

Refinement on *F*^2^	Primary atom site location: structure-invariant direct methods
Least-squares matrix: full	Secondary atom site location: difference Fourier map
*R*[*F*^2^ > 2σ(*F*^2^)] = 0.051	Hydrogen site location: inferred from neighbouring sites
*wR*(*F*^2^) = 0.143	H-atom parameters constrained
*S* = 1.02	*w* = 1/[σ^2^(*F*_o_^2^) + (0.055*P*)^2^ + 1.1784*P*] where *P* = (*F*_o_^2^ + 2*F*_c_^2^)/3
5960 reflections	(Δ/σ)_max_ < 0.001
298 parameters	Δρ_max_ = 0.51 e Å^−^^3^
0 restraints	Δρ_min_ = −0.66 e Å^−^^3^

### Special details

**Table:**

Geometry. All e.s.d.'s (except the e.s.d. in the dihedral angle between two l.s. planes) are estimated using the full covariance matrix. The cell e.s.d.'s are taken into account individually in the estimation of e.s.d.'s in distances, angles and torsion angles; correlations between e.s.d.'s in cell parameters are only used when they are defined by crystal symmetry. An approximate (isotropic) treatment of cell e.s.d.'s is used for estimating e.s.d.'s involving l.s. planes.
Refinement. Refinement of *F*^2^ against ALL reflections. The weighted *R*-factor *wR* and goodness of fit *S* are based on *F*^2^, conventional *R*-factors *R* are based on *F*, with *F* set to zero for negative *F*^2^. The threshold expression of *F*^2^ > σ(*F*^2^) is used only for calculating *R*-factors(gt) *etc*. and is not relevant to the choice of reflections for refinement. *R*-factors based on *F*^2^ are statistically about twice as large as those based on *F*, and *R*- factors based on ALL data will be even larger.

### Fractional atomic coordinates and isotropic or equivalent isotropic displacement parameters (Å^2^)

**Table:**

	*x*	*y*	*z*	*U*_iso_*/*U*_eq_	
C1	0.0838 (3)	0.35507 (15)	0.51570 (10)	0.0529 (5)	
C2	0.0846 (4)	0.30775 (17)	0.57843 (12)	0.0673 (7)	
H2	0.1872	0.3031	0.6041	0.081*	
C3	−0.0648 (4)	0.26917 (18)	0.60111 (13)	0.0744 (8)	
H3	−0.0624	0.2371	0.6418	0.089*	
C4	−0.2209 (4)	0.27669 (17)	0.56474 (14)	0.0713 (8)	
H4	−0.3214	0.2498	0.5814	0.086*	
C5	−0.2280 (4)	0.32318 (16)	0.50486 (13)	0.0645 (6)	
H5	−0.3332	0.3283	0.4809	0.077*	
C6	−0.0756 (3)	0.36356 (14)	0.47927 (11)	0.0514 (5)	
C7	−0.0714 (3)	0.41213 (14)	0.41763 (11)	0.0487 (5)	
H7	−0.1739	0.4215	0.3928	0.058*	
C8	0.0829 (3)	0.44546 (13)	0.39442 (9)	0.0412 (4)	
C9	0.2379 (3)	0.43039 (14)	0.43295 (9)	0.0447 (5)	
C10	0.4114 (3)	0.45889 (15)	0.40672 (10)	0.0500 (5)	
H10A	0.4716	0.4077	0.3886	0.060*	
H10B	0.4804	0.4812	0.4449	0.060*	
C11	0.4037 (3)	0.52926 (14)	0.35131 (10)	0.0467 (5)	
H11A	0.3775	0.5857	0.3723	0.056*	
H11B	0.5173	0.5339	0.3298	0.056*	
C12	0.2668 (2)	0.50947 (12)	0.29669 (9)	0.0370 (4)	
C13	0.0870 (3)	0.49484 (13)	0.32843 (9)	0.0403 (4)	
C14	0.2591 (2)	0.57197 (12)	0.23452 (9)	0.0368 (4)	
H14	0.1517	0.6072	0.2358	0.044*	
C15	0.2486 (2)	0.50547 (12)	0.17730 (9)	0.0387 (4)	
C16	0.2293 (3)	0.52867 (13)	0.10425 (10)	0.0438 (4)	
C17	0.2685 (4)	0.46792 (16)	0.05371 (11)	0.0595 (6)	
H17	0.3050	0.4116	0.0662	0.071*	
C18	0.2543 (4)	0.48944 (18)	−0.01475 (11)	0.0668 (7)	
H18	0.2803	0.4480	−0.0484	0.080*	
C19	0.2013 (3)	0.57288 (18)	−0.03267 (11)	0.0604 (6)	
C20	0.1643 (3)	0.63492 (18)	0.01599 (12)	0.0635 (6)	
H20	0.1302	0.6914	0.0030	0.076*	
C21	0.1782 (3)	0.61285 (16)	0.08492 (11)	0.0545 (5)	
H21	0.1530	0.6547	0.1183	0.065*	
C22	0.4159 (2)	0.63149 (12)	0.22639 (9)	0.0380 (4)	
C23	0.4050 (3)	0.72015 (13)	0.24167 (10)	0.0449 (4)	
H23	0.2985	0.7442	0.2548	0.054*	
C24	0.5513 (3)	0.77360 (14)	0.23758 (11)	0.0528 (5)	
H24	0.5440	0.8334	0.2479	0.063*	
C25	0.7065 (3)	0.73722 (16)	0.21810 (11)	0.0532 (6)	
C26	0.7203 (3)	0.64889 (16)	0.20123 (12)	0.0561 (6)	
H26	0.8266	0.6253	0.1874	0.067*	
C27	0.5743 (3)	0.59690 (14)	0.20536 (11)	0.0488 (5)	
H27	0.5817	0.5375	0.1939	0.059*	
N1	0.2385 (3)	0.38799 (13)	0.49232 (8)	0.0536 (5)	
N2	0.2748 (2)	0.42569 (11)	0.19573 (8)	0.0430 (4)	
O1	−0.0456 (2)	0.51723 (12)	0.29909 (8)	0.0617 (4)	
O2	0.30472 (18)	0.42271 (8)	0.26726 (6)	0.0424 (3)	
Cl1	0.18361 (12)	0.59980 (6)	−0.11902 (3)	0.0901 (3)	
Cl2	0.89238 (10)	0.80315 (5)	0.21560 (4)	0.0828 (3)	

### Atomic displacement parameters (Å^2^)

**Table:**

	*U*^11^	*U*^22^	*U*^33^	*U*^12^	*U*^13^	*U*^23^
C1	0.0719 (15)	0.0483 (12)	0.0386 (10)	0.0143 (10)	0.0080 (10)	0.0029 (8)
C2	0.091 (2)	0.0665 (16)	0.0440 (11)	0.0183 (14)	0.0048 (12)	0.0111 (11)
C3	0.109 (2)	0.0636 (16)	0.0510 (13)	0.0186 (15)	0.0246 (15)	0.0167 (11)
C4	0.090 (2)	0.0566 (15)	0.0682 (16)	0.0104 (14)	0.0296 (15)	0.0166 (12)
C5	0.0696 (16)	0.0603 (15)	0.0638 (14)	0.0094 (12)	0.0178 (12)	0.0145 (11)
C6	0.0639 (14)	0.0440 (11)	0.0464 (10)	0.0117 (10)	0.0131 (10)	0.0054 (9)
C7	0.0501 (12)	0.0489 (12)	0.0471 (10)	0.0093 (9)	0.0046 (9)	0.0078 (9)
C8	0.0470 (11)	0.0410 (10)	0.0355 (8)	0.0083 (8)	0.0027 (8)	0.0008 (7)
C9	0.0520 (12)	0.0475 (11)	0.0345 (9)	0.0096 (9)	−0.0014 (8)	−0.0033 (8)
C10	0.0476 (12)	0.0667 (14)	0.0355 (9)	0.0069 (10)	−0.0093 (8)	−0.0007 (9)
C11	0.0443 (11)	0.0532 (12)	0.0424 (10)	−0.0016 (9)	−0.0057 (8)	−0.0032 (9)
C12	0.0387 (10)	0.0352 (9)	0.0369 (8)	0.0038 (7)	−0.0024 (7)	−0.0002 (7)
C13	0.0415 (11)	0.0414 (10)	0.0381 (9)	0.0043 (8)	−0.0010 (8)	0.0022 (7)
C14	0.0356 (10)	0.0346 (9)	0.0400 (9)	0.0017 (7)	−0.0016 (7)	0.0009 (7)
C15	0.0395 (10)	0.0386 (10)	0.0379 (9)	−0.0043 (8)	−0.0016 (7)	0.0009 (7)
C16	0.0460 (11)	0.0468 (11)	0.0385 (9)	−0.0093 (9)	−0.0029 (8)	0.0045 (8)
C17	0.0833 (18)	0.0516 (13)	0.0435 (11)	−0.0079 (12)	−0.0013 (11)	0.0003 (9)
C18	0.0899 (19)	0.0700 (16)	0.0403 (11)	−0.0166 (14)	0.0019 (11)	−0.0052 (10)
C19	0.0641 (15)	0.0789 (17)	0.0382 (10)	−0.0215 (13)	−0.0078 (10)	0.0109 (10)
C20	0.0731 (16)	0.0661 (15)	0.0511 (12)	−0.0023 (13)	−0.0066 (11)	0.0175 (11)
C21	0.0635 (14)	0.0571 (13)	0.0430 (10)	0.0016 (11)	−0.0023 (10)	0.0060 (9)
C22	0.0409 (10)	0.0351 (9)	0.0380 (9)	−0.0011 (8)	−0.0025 (7)	0.0016 (7)
C23	0.0503 (12)	0.0382 (10)	0.0462 (10)	0.0016 (8)	−0.0012 (9)	0.0003 (8)
C24	0.0674 (15)	0.0393 (11)	0.0515 (11)	−0.0114 (10)	−0.0097 (10)	0.0036 (9)
C25	0.0511 (13)	0.0591 (14)	0.0491 (11)	−0.0207 (10)	−0.0135 (9)	0.0163 (10)
C26	0.0395 (12)	0.0659 (15)	0.0629 (13)	−0.0025 (10)	0.0002 (10)	0.0089 (11)
C27	0.0432 (11)	0.0414 (11)	0.0617 (12)	0.0006 (9)	0.0020 (9)	−0.0020 (9)
N1	0.0654 (12)	0.0597 (11)	0.0358 (8)	0.0118 (9)	−0.0019 (8)	0.0041 (8)
N2	0.0510 (10)	0.0412 (9)	0.0368 (8)	−0.0002 (7)	−0.0032 (7)	−0.0015 (6)
O1	0.0410 (9)	0.0822 (12)	0.0620 (9)	0.0053 (8)	−0.0031 (7)	0.0296 (8)
O2	0.0531 (8)	0.0377 (7)	0.0365 (6)	0.0092 (6)	−0.0028 (6)	0.0010 (5)
Cl1	0.1173 (7)	0.1131 (6)	0.0398 (3)	−0.0268 (5)	−0.0110 (3)	0.0189 (3)
Cl2	0.0693 (4)	0.0932 (5)	0.0855 (5)	−0.0443 (4)	−0.0209 (4)	0.0288 (4)

### Geometric parameters (Å, º)

**Table:**

C1—N1	1.365 (3)	C14—C15	1.504 (2)
C1—C6	1.413 (3)	C14—C22	1.510 (3)
C1—C2	1.417 (3)	C14—H14	0.9800
C2—C3	1.360 (4)	C15—N2	1.277 (2)
C2—H2	0.9300	C15—C16	1.472 (3)
C3—C4	1.389 (4)	C16—C17	1.382 (3)
C3—H3	0.9300	C16—C21	1.386 (3)
C4—C5	1.363 (3)	C17—C18	1.376 (3)
C4—H4	0.9300	C17—H17	0.9300
C5—C6	1.411 (3)	C18—C19	1.372 (4)
C5—H5	0.9300	C18—H18	0.9300
C6—C7	1.409 (3)	C19—C20	1.366 (4)
C7—C8	1.364 (3)	C19—Cl1	1.735 (2)
C7—H7	0.9300	C20—C21	1.387 (3)
C8—C9	1.417 (3)	C20—H20	0.9300
C8—C13	1.488 (3)	C21—H21	0.9300
C9—N1	1.323 (2)	C22—C23	1.379 (3)
C9—C10	1.490 (3)	C22—C27	1.385 (3)
C10—C11	1.518 (3)	C23—C24	1.384 (3)
C10—H10A	0.9700	C23—H23	0.9300
C10—H10B	0.9700	C24—C25	1.365 (3)
C11—C12	1.518 (3)	C24—H24	0.9300
C11—H11A	0.9700	C25—C26	1.383 (3)
C11—H11B	0.9700	C25—Cl2	1.739 (2)
C12—O2	1.464 (2)	C26—C27	1.370 (3)
C12—C13	1.528 (3)	C26—H26	0.9300
C12—C14	1.538 (2)	C27—H27	0.9300
C13—O1	1.209 (2)	N2—O2	1.4111 (19)
			
N1—C1—C6	123.12 (18)	C15—C14—C22	111.14 (15)
N1—C1—C2	118.3 (2)	C15—C14—C12	99.93 (14)
C6—C1—C2	118.5 (2)	C22—C14—C12	115.11 (15)
C3—C2—C1	119.9 (3)	C15—C14—H14	110.1
C3—C2—H2	120.0	C22—C14—H14	110.1
C1—C2—H2	120.0	C12—C14—H14	110.1
C2—C3—C4	121.4 (2)	N2—C15—C16	120.84 (17)
C2—C3—H3	119.3	N2—C15—C14	114.74 (16)
C4—C3—H3	119.3	C16—C15—C14	124.10 (16)
C5—C4—C3	120.5 (3)	C17—C16—C21	118.80 (19)
C5—C4—H4	119.7	C17—C16—C15	120.57 (19)
C3—C4—H4	119.7	C21—C16—C15	120.58 (19)
C4—C5—C6	119.9 (3)	C18—C17—C16	121.1 (2)
C4—C5—H5	120.1	C18—C17—H17	119.5
C6—C5—H5	120.1	C16—C17—H17	119.5
C7—C6—C5	123.5 (2)	C19—C18—C17	119.1 (2)
C7—C6—C1	116.8 (2)	C19—C18—H18	120.5
C5—C6—C1	119.7 (2)	C17—C18—H18	120.5
C8—C7—C6	120.1 (2)	C20—C19—C18	121.3 (2)
C8—C7—H7	120.0	C20—C19—Cl1	119.7 (2)
C6—C7—H7	120.0	C18—C19—Cl1	118.9 (2)
C7—C8—C9	119.17 (18)	C19—C20—C21	119.4 (2)
C7—C8—C13	119.74 (18)	C19—C20—H20	120.3
C9—C8—C13	121.07 (18)	C21—C20—H20	120.3
N1—C9—C8	122.6 (2)	C16—C21—C20	120.3 (2)
N1—C9—C10	116.29 (18)	C16—C21—H21	119.9
C8—C9—C10	121.05 (17)	C20—C21—H21	119.9
C9—C10—C11	114.63 (17)	C23—C22—C27	119.15 (19)
C9—C10—H10A	108.6	C23—C22—C14	120.67 (18)
C11—C10—H10A	108.6	C27—C22—C14	120.15 (17)
C9—C10—H10B	108.6	C22—C23—C24	120.5 (2)
C11—C10—H10B	108.6	C22—C23—H23	119.8
H10A—C10—H10B	107.6	C24—C23—H23	119.8
C12—C11—C10	112.53 (17)	C25—C24—C23	119.0 (2)
C12—C11—H11A	109.1	C25—C24—H24	120.5
C10—C11—H11A	109.1	C23—C24—H24	120.5
C12—C11—H11B	109.1	C24—C25—C26	121.7 (2)
C10—C11—H11B	109.1	C24—C25—Cl2	119.25 (19)
H11A—C11—H11B	107.8	C26—C25—Cl2	119.06 (19)
O2—C12—C11	108.33 (15)	C27—C26—C25	118.6 (2)
O2—C12—C13	102.09 (14)	C27—C26—H26	120.7
C11—C12—C13	111.31 (15)	C25—C26—H26	120.7
O2—C12—C14	104.56 (13)	C26—C27—C22	121.0 (2)
C11—C12—C14	116.92 (16)	C26—C27—H27	119.5
C13—C12—C14	112.23 (15)	C22—C27—H27	119.5
O1—C13—C8	121.80 (18)	C9—N1—C1	118.12 (19)
O1—C13—C12	121.48 (17)	C15—N2—O2	109.40 (15)
C8—C13—C12	116.55 (16)	N2—O2—C12	109.06 (12)
			
N1—C1—C2—C3	−175.8 (2)	C12—C14—C15—N2	−9.7 (2)
C6—C1—C2—C3	2.2 (3)	C22—C14—C15—C16	−61.2 (2)
C1—C2—C3—C4	−1.5 (4)	C12—C14—C15—C16	176.79 (18)
C2—C3—C4—C5	0.1 (4)	N2—C15—C16—C17	−11.1 (3)
C3—C4—C5—C6	0.4 (4)	C14—C15—C16—C17	162.0 (2)
C4—C5—C6—C7	179.5 (2)	N2—C15—C16—C21	171.5 (2)
C4—C5—C6—C1	0.4 (4)	C14—C15—C16—C21	−15.4 (3)
N1—C1—C6—C7	−2.9 (3)	C21—C16—C17—C18	−1.2 (4)
C2—C1—C6—C7	179.1 (2)	C15—C16—C17—C18	−178.6 (2)
N1—C1—C6—C5	176.2 (2)	C16—C17—C18—C19	0.4 (4)
C2—C1—C6—C5	−1.7 (3)	C17—C18—C19—C20	0.7 (4)
C5—C6—C7—C8	−176.7 (2)	C17—C18—C19—Cl1	180.0 (2)
C1—C6—C7—C8	2.5 (3)	C18—C19—C20—C21	−0.9 (4)
C6—C7—C8—C9	0.1 (3)	Cl1—C19—C20—C21	179.79 (19)
C6—C7—C8—C13	178.54 (18)	C17—C16—C21—C20	0.9 (3)
C7—C8—C9—N1	−2.7 (3)	C15—C16—C21—C20	178.4 (2)
C13—C8—C9—N1	178.90 (18)	C19—C20—C21—C16	0.1 (4)
C7—C8—C9—C10	174.79 (19)	C15—C14—C22—C23	141.26 (18)
C13—C8—C9—C10	−3.6 (3)	C12—C14—C22—C23	−106.1 (2)
N1—C9—C10—C11	−162.66 (18)	C15—C14—C22—C27	−40.7 (2)
C8—C9—C10—C11	19.7 (3)	C12—C14—C22—C27	72.0 (2)
C9—C10—C11—C12	−45.5 (2)	C27—C22—C23—C24	−1.4 (3)
C10—C11—C12—O2	−56.8 (2)	C14—C22—C23—C24	176.62 (18)
C10—C11—C12—C13	54.6 (2)	C22—C23—C24—C25	0.1 (3)
C10—C11—C12—C14	−174.55 (16)	C23—C24—C25—C26	1.2 (3)
C7—C8—C13—O1	10.5 (3)	C23—C24—C25—Cl2	−177.88 (16)
C9—C8—C13—O1	−171.1 (2)	C24—C25—C26—C27	−1.0 (3)
C7—C8—C13—C12	−164.82 (18)	Cl2—C25—C26—C27	178.05 (17)
C9—C8—C13—C12	13.6 (3)	C25—C26—C27—C22	−0.4 (3)
O2—C12—C13—O1	−98.6 (2)	C23—C22—C27—C26	1.6 (3)
C11—C12—C13—O1	146.0 (2)	C14—C22—C27—C26	−176.45 (19)
C14—C12—C13—O1	12.8 (3)	C8—C9—N1—C1	2.3 (3)
O2—C12—C13—C8	76.68 (18)	C10—C9—N1—C1	−175.28 (19)
C11—C12—C13—C8	−38.7 (2)	C6—C1—N1—C9	0.6 (3)
C14—C12—C13—C8	−171.91 (16)	C2—C1—N1—C9	178.5 (2)
O2—C12—C14—C15	13.95 (17)	C16—C15—N2—O2	174.59 (16)
C11—C12—C14—C15	133.71 (17)	C14—C15—N2—O2	0.9 (2)
C13—C12—C14—C15	−95.92 (17)	C15—N2—O2—C12	9.2 (2)
O2—C12—C14—C22	−105.16 (17)	C11—C12—O2—N2	−140.22 (15)
C11—C12—C14—C22	14.6 (2)	C13—C12—O2—N2	102.23 (15)
C13—C12—C14—C22	144.98 (16)	C14—C12—O2—N2	−14.85 (18)
C22—C14—C15—N2	112.27 (19)		

### Hydrogen-bond geometry (Å, º)

**Table:**

*D*—H···*A*	*D*—H	H···*A*	*D*···*A*	*D*—H···*A*
C10—H10*A*···Cl2^i^	0.97	2.78	3.681 (2)	154

Symmetry code: (i) −*x*+3/2, *y*−1/2, −*z*+1/2.
